# The Effect of Ball Burnishing on Tribological Performance of 42CrMo4 Steel under Dry Sliding Conditions

**DOI:** 10.3390/ma13092127

**Published:** 2020-05-03

**Authors:** Slawomir Swirad, Pawel Pawlus

**Affiliations:** Faculty of Mechanical Engineering and Aeronautics, Rzeszow University of Technology, Powstancow Warszawy 8 Street, 35-959 Rzeszow, Poland; s.swirad@prz.edu.pl

**Keywords:** ball burnishing, coefficient of friction, volumetric wear

## Abstract

Ball burnishing appears to be a very promising approach for reducing surface height, generating compressive residual stresses and increasing hardness. Ball burnishing treatment was carried out using a Haas CNC Vertical Mill Center VF-1 equipped with the Ecoroll burnishing system. After burnishing, surface topographies of machined samples and hardness were measured. Wear tests were conducted using a ball-on-disc tribotester in dry sliding conditions. During tests, the friction force was monitored as a function of time. After tests, wear volumes were determined on the basis of surface texture measurements. Tests revealed that ball burnishing in most cases resulted in minimizing friction and wear of contacting elements.

## 1. Introduction 

In the manufacturing industry, two components are very important: the dimensional accuracy and the quality of a surface finish. They can assure achievements of the proper functional performances of machine elements.

Machining hard materials has been a challenge in many industries such as automotive, aviation, hydraulic as well as die and mold making [1,2]. Grinding or other kinds of abrasive treatment (e.g., honing) are the most common finishing processes of steel and other materials. Recently, attempts were made to eliminate or limit this type of processing in favor of loss-free smoothing treatments such as ball burnishing. Height of surface topography can be significantly reduced by application of superfinishing and burnishing after hard turning [3]. The process of ball burnishing is typically performed with a ball (very smooth and hardened) which slides over the surface of the processed material, and, at the same time, is pressed against it with a normal force [4]. In the contact zone, the normal force generates contact stress in the material surface layer. If the stress is higher than the material’s yield strength, the material near the surface starts to flow. Due to the plastic deformation (material flow) of the surface layer, a very smooth surface can be achieved. Swirad et al. obtained substantial reduction in the height standard deviation using the raster strategy of ball burnishing [5]. During the burnishing process, the reduction of surface roughness amplitude, the increase of the surface layer hardness and the introduction of compressive residual stress to a depth near 1 mm are obtained, which enhance surface layer integrity [6]. 

In technical literature, the studies considering the effect of parameters of the ball burnishing process on the surface texture are frequently found. The input parameters typically include a burnishing force, a feed, a burnishing speed, a burnishing width, and a pass number. Swirad [7] changed the burnishing rate and the applied pressure, while Dzionk et al. [8] changed the feed and the burnishing speed. The ball burnishing process can be used in a wide range of materials from steel through titanium [9] and aluminum to magnesium alloys [10] and coated surfaces [11]. 

The influences of the input parameters of the burnishing process on the texture of magnesium alloy surfaces AZ91 and AZ91D were analyzed [12,13]. Korzynski and Zarski [12] used a burnishing tool for sliding burnishing. As a result of this process, a reduction in the surface texture amplitude defined by the arithmetic mean height Sa from 1.6 to 0.4 µm was achieved. The burnishing force and the tool diameter were the most crucial parameters of the process. Buldum and Cagan [13] obtained a reduction in the arithmetic mean height to 0.336 µm. Cobanoglu et al. [14] achieved a reduction in the Ra parameter of steel AISI 1040 from 0.45 to 0.13 µm. Revankar et al. [15] applied a burnishing process for a titanium alloy Ti-6Al-4V. The study revealed that the surface roughness height was reduced by 77%, while microhardness increased from 340 to 406 HV due to burnishing. Furthermore, it was found that after the burnishing process in the surface layer, compressive residual stresses were developed in axial and tangential directions of about −800 MPa and at the depth of 160 µm. Additionally, this kind of a lubricant was proved to have a crucial impact on the process. A decrease in volumetric wear after ball burnishing is typically related to an increase in hardness [16,17]. Revankar et al. [16] showed that application of Taguchi optimization resulted in high improvements in a specific wear rate (52% decrease) and the coefficient of friction (64% decrease) of titanium alloy Ti-6Al-4V during ball burnishing. Travieso-Rodriguez et al. [17] analyzed the influence of ball burnishing on the hardening effect and fatigue behavior of steel AISI 1038. They observed that hardness increased when the degree of plastic deformation increased. It was possible to increase the number of cycles for the burnishing force of 120 N, 7 passes with a 6 mm ball diameter. Similar results were obtained after shot peening [18,19]. An improvement in the wear resistance was caused by increased hardness in comparison to ground surfaces [18]. Xue et al. [19] obtained the wear reduction up to 73% due to shot peening. 

The improvement of tribological properties due to ball burnishing can be achieved due to changes in the surface topography. Whitehouse [20] and Petropoulos et al. [21] indicated the suitability of the parameters of peaks and valleys, as well as the skewness and kurtosis in the evaluation of friction and wear. The skewness Ssk describes the symmetry of the surface texture. A negative value of skewness indicates that the surface has mainly valleys, whereas a surface with a positive skewness contains primarily peaks. The kurtosis Sku characterizes the sharpness of the surface probability distribution. Belhadjamor et al. [22] and Zhan et al. [23] found beneficial effects of negatively skewed surfaces. A negative skewness increased the normal contact stiffness [22] and led to smaller mean separation for a given normal load and higher tangential stiffness, however, different trends were observed for positive skewness [23]. The negative skewness can improve the contact and sliding conditions of smooth textures [24]. Similar results were obtained in experimental investigations under dry sliding conditions [25,26,27,28]. Negative values of the Ssk parameter led to reductions in wear volumes of smooth [25] and rough [26] steel discs. Sedlacek at. al. [27] found that negative values of skewness Ssk led to the friction reduction. Two-process surfaces, having traces of two machining processes, are highly skewed structures. These surfaces can lead to better tribological performance compared to one-process textures; these beneficial effects depend on the operating conditions [28,29]. However, other methods of characterization of surface topography important in tribological problems exist. For example, recently, an article was published using 3D reconstruction by extended depth-of-field reconstruction to evaluate the tribological performance of PA66 composites, using the fractal approach [30].

Ball burnishing is a promising approach for reducing surface height, generating compressive residual stresses and increasing hardness. It should also cause improvement of tribological properties of contacting elements. However, there is only a limited number of research works concerning the effect of ball burnishing on friction and wear of sliding pairs. The present authors attempt to fill this gap.

The purpose of this work is to study the effect of ball burnishing on the tribological performance of the sliding pair in dry sliding conditions under ball-on-disc configuration. The burnishing pressure was the input parameter. After burnishing, surface topography and hardness were measured. 

## 2. Experimental Details

Ball burnishing tests were conducted using the Haas CNC Vertical Mill Center VF-1 (Haas Automation Inc., Oxnard, CA, USA) equipped with the Ecoroll (Ecoroll AG Werkzeugtechnik, Celle, Germany) burnishing system. The burnishing system consists of a high-pressure pump and a burnishing tool of 6 mm diameter, which are connected via pressure hoses. The pressure was provided by a hydraulic pump. The maximum pressure of 40 MPa can be generated. The following burnishing pressures P were used in the present tests: 10, 20, 30 and 40 MPa. A burnishing strategy and other parameters such as the burnishing speed v and the width remained constant. The machining parameters are shown in Table 1. 

The material used in this study was 42CrMo4 chromium–molybdenum alloy steel of hardness 34 ± 2 HRC. The burnished samples had a height of 9.0 mm and a diameter of 25.4 mm, therefore, they could be used for wear tests.

Before burnishing, samples were milled. The milling parameters were as follows: the rotational speed n=950 rev/min, the feed per tooth fz = 0.1 mm/tooth, the feed speed vf = 400 mm/min, the depth of cut ap = 0.2 mm. The cutting speed vc was calculated using the effective cutting diameter deff. For ap = 0.2 mm, deff = 2.62 mm, and therefore, vc = 100 m/min. 

Measurement of surface topography parameters was conducted after ball burnishing and after tribological tests. For this purpose, a white light interferometer Talysurf CCI Lite (Taylor Hobson Ltd Leicester, UK) with a vertical resolution of 0.01 nm was used. The measurement area was 3.3 × 3.3 mm^2^. Measured textures were only leveled without digital filtration. The surface topography parameters were calculated using the TalyMap (Digital Surf, Besancon, France) software.

Surface hardness represents the degree of plastic deformation caused on the specimen. It was measured through Vickers microindentation tests by applying 3 N load. Microhardness tester Reicherter Brivisor KL2 (Buehler Ltd., Lake Bluff, IL, USA) with a lens system was the equipment. Five indentations (trials) at each workpiece were performed. The load duration was 20 s. The magnification of the device measuring the dimensions of indentations was 400 ×.

Wear tests were conducted using a ball-on-disc tribotester in dry sliding conditions (model T-11, Institute for Sustainable Technologies, Radom, Poland). Burnished discs from 42CrMo4 steel were put in contact with 100Cr6 steel balls of 60 HRC hardness. The tests were carried out at a sliding speed of 0.36 m/s at ambient temperature (20–22 °C), the relative humidity was 40–50%. The test durations were set to 10 minutes, and the normal forces were 10, 20 and 30 N. The number of test repetitions was 3. The friction radius was 5 mm. 

During each test, the friction force was monitored as a function of time. Wear of discs was determined by means of surface topography analysis. The profiles, orthogonal to wear track, taken in four positions (90° apart) were processed to obtain the mean wear scar area, which was multiplied by the track length to obtain volumetric wear. 

**Figure 1:**
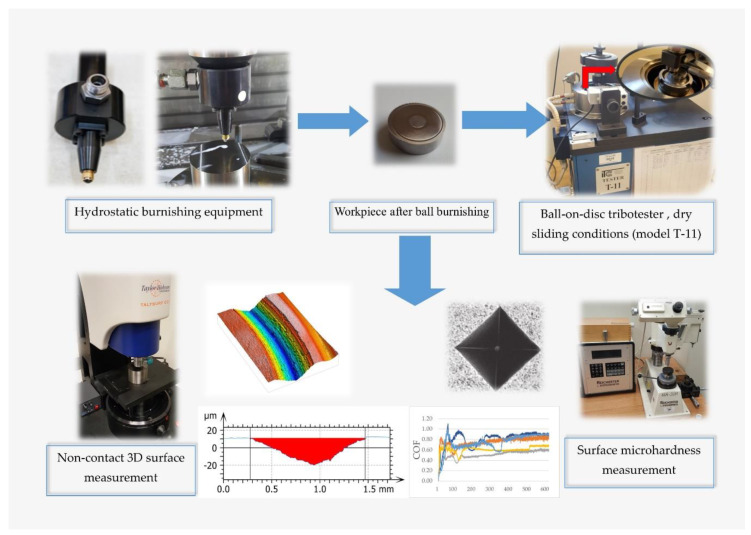
The schematic diagram of the experimental arrangement.

A schematic diagram of the experimental arrangement is shown in Figure 1.

## 3. Results and Discussion

**Figure 2:**
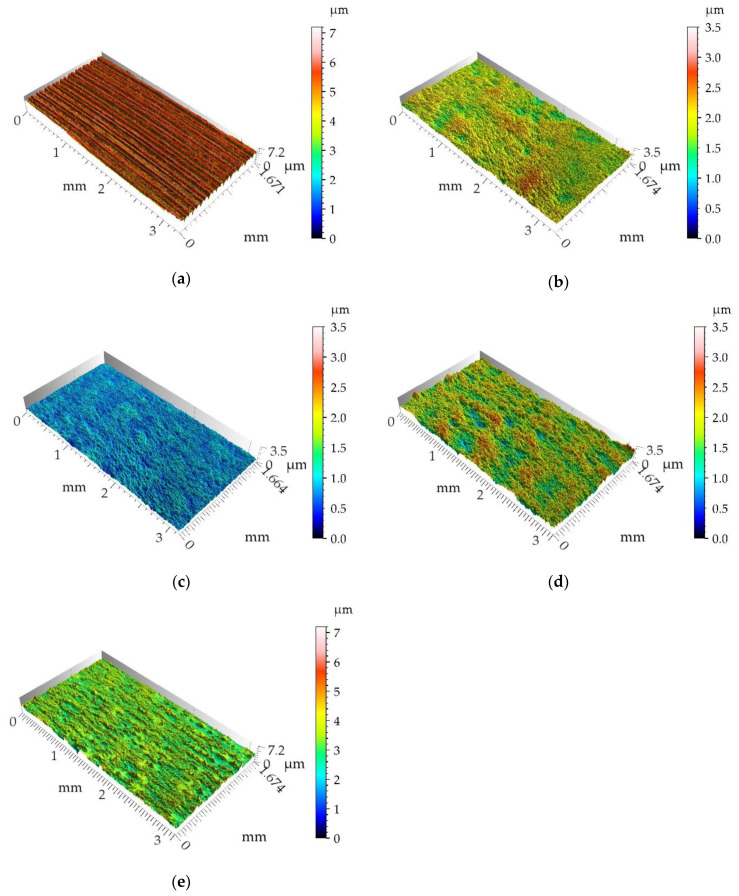
Isometric views of disc samples after milling (**a**), after burnishing with pressure of 10 (**b**), 20 (**c**), 30 (**d**) and 40 MPa (**e**).

**Figure 3:**
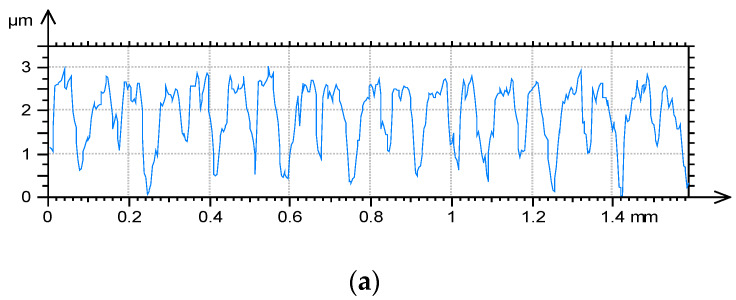

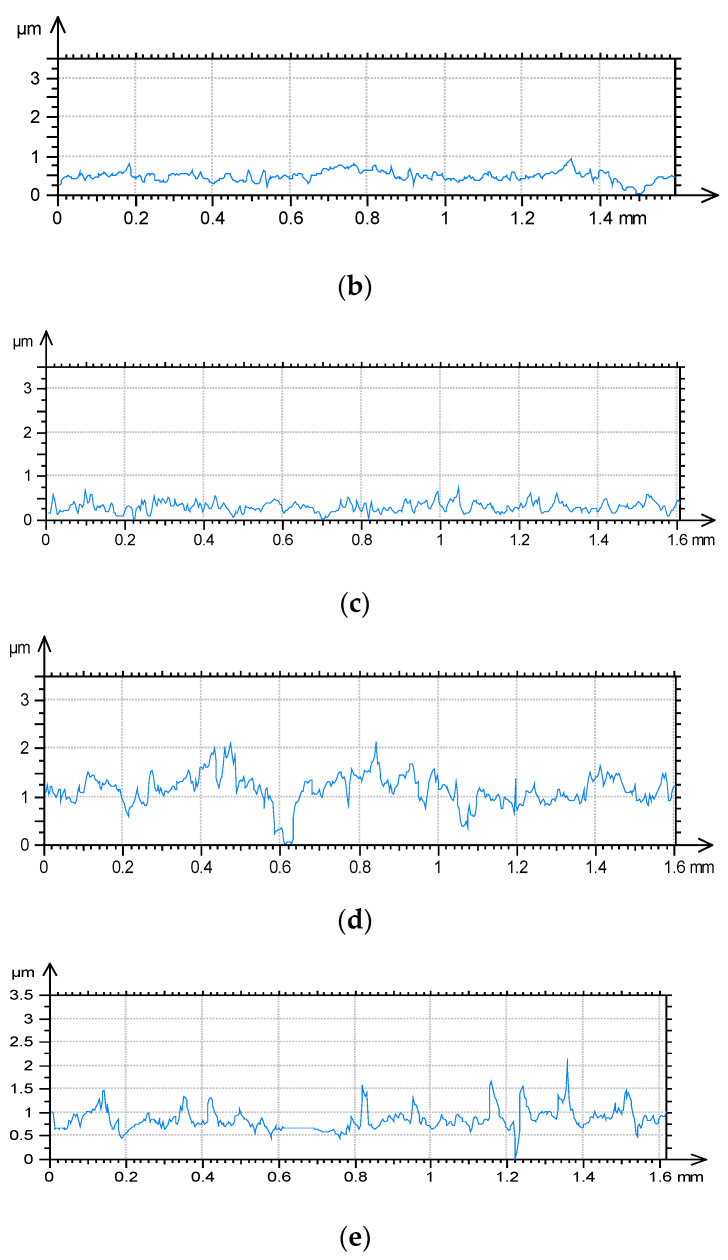
Selected profiles of disc samples after milling (**a**), burnishing with pressure of 10 (**b**), 20 (**c**), 30 (**d**) and 40 MPa (**e**).

Figure 2 shows isometric views, while Figure 3 selected profiles of the tested disc samples. Table 2 presents selected surface topography parameters of disc specimens according to the ISO 25411 standard. The surface after milling is one-process deterministic one-directional anisotropic texture of the asymmetric ordinate distribution (the skewness Sku is −0.71). The value of kurtosis (2.64) is characteristic for the deterministic texture. A very small value of the Str parameter proves of the anisotropic character of the milled surface topography. Due to burnishing, amplitude parameters (rms. height Sq, maximum peak height Sp, maximum valley depth Sv, total surface height Sz and arithmetical mean height Sa) and hybrid parameters (rms. slope Sdq and developed interfacial area ratio Sdr) typically decreased. This reduction was the highest, when the burnishing pressure 20 MPa was applied followed by the pressure of 10 MPa. From among burnished discs, the biggest height and hybrid parameters were obtained for the disc surfaces machined with the highest burnishing pressures of 30 and 40 MPa. From among these surfaces, the texture obtained after using the highest pressure (40 MPa) was characterized by higher values of skewness Ssk (0.91) and kurtosis Sku (7.3), a smaller value of the correlation length Sal (0.028 mm), the texture-aspect ratio (0.22) and the peak density Spd (354.7 1/µm^2^). Burnishing caused also increases in Sal and Str, and typically Spd parameters, and a decrease in the arithmetic mean peak curvature Spc. Generally, the burnished surfaces were one-process random mixed (Str between 0.22 and 0.43) structures.

**Figure 4:**
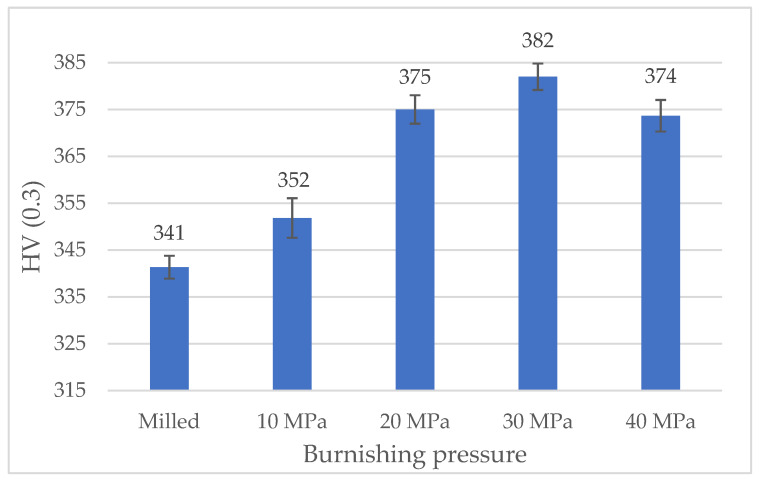
The results of hardness measurement of disc samples.

Figure 4 presents the results of hardness measurement of disc samples. Ball burnishing caused an increase in hardness. This growth was the lowest when burnishing pressure was the smallest (10 MPa). Similar hardness increases were achieved for higher pressures, and the largest value was obtained when P = 30 MPa.

**Figure 5:**
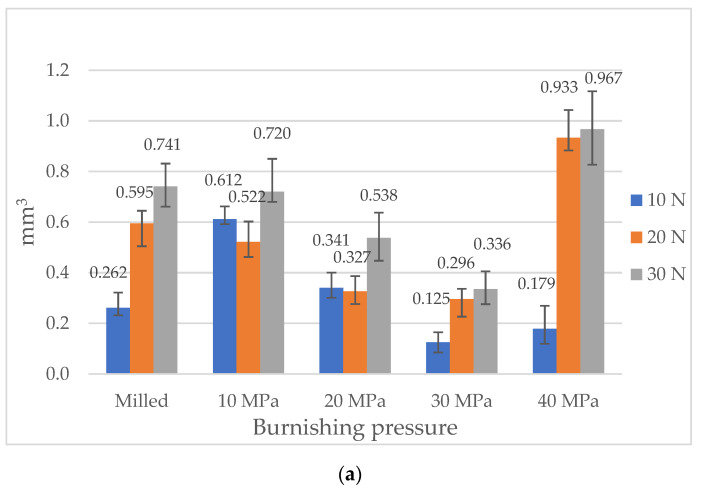

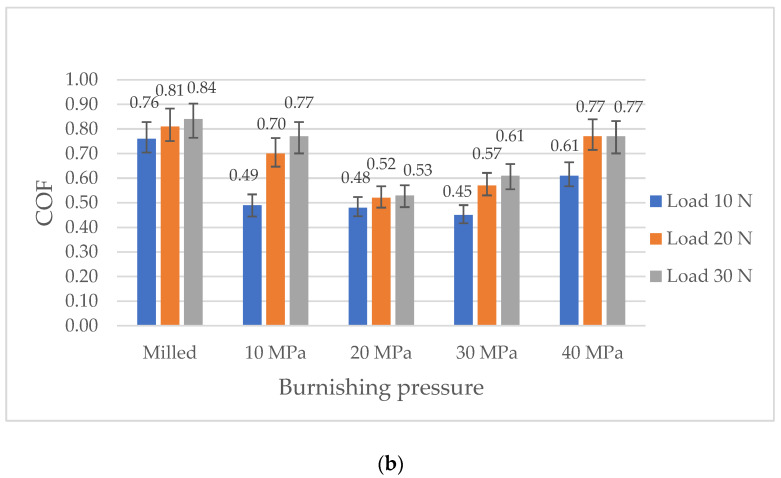
The results of wear examination of disc samples (**a**) and mean values of the coefficient of friction for tested sliding pairs (**b**).

Figure 5 presents the results of the wear examination of tested disc samples and mean values of the coefficient of friction. Wear levels of balls were negligible due to the difference in hardness between two counterparts. The mean coefficients of friction were obtained after 100 s to exclude initial fluctuations. 

**Figure 6:**
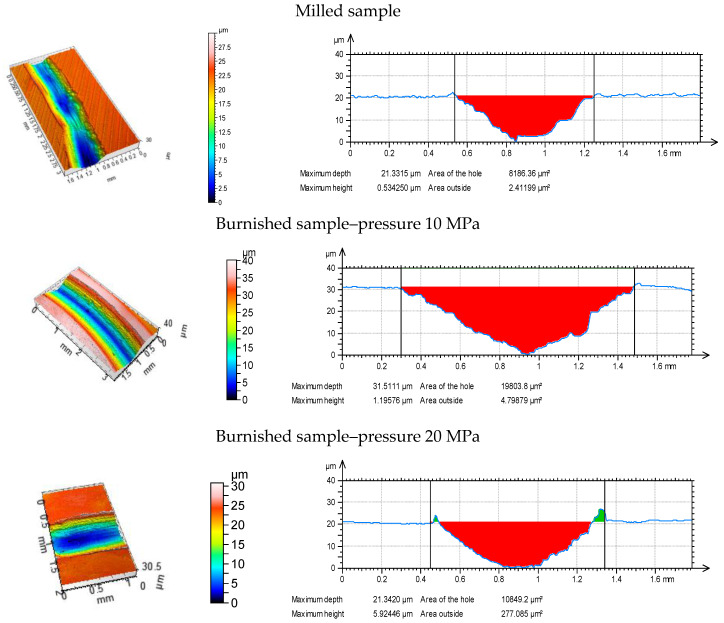

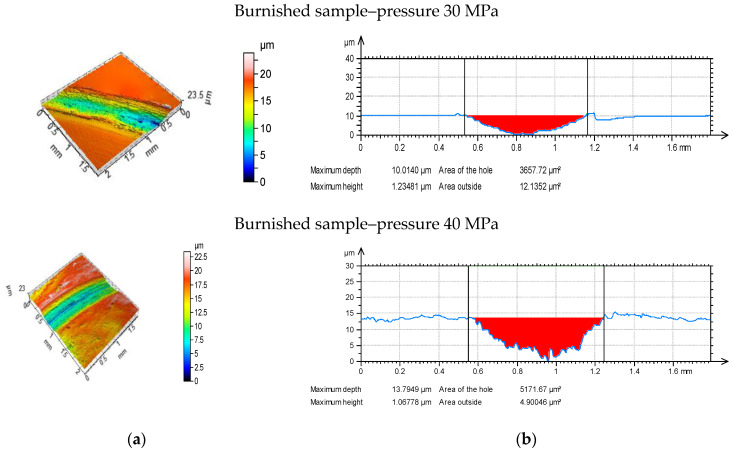
Isometric views (**a**) and profiles perpendicular to wear scars (**b**) for the tested disc samples at normal load of 10 N.

**Figure 7:**
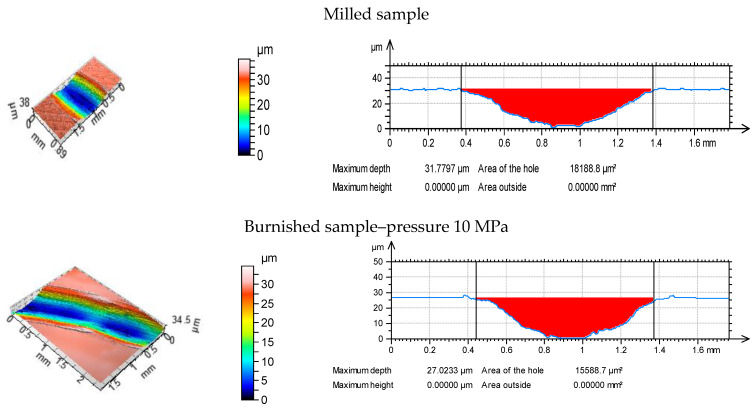

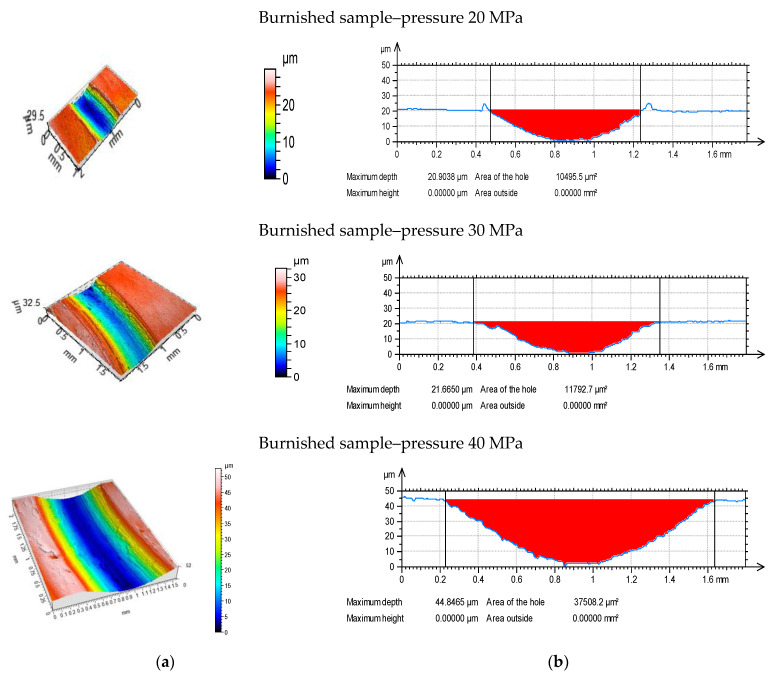
Isometric views (**a**) and profiles perpendicular to wear scars (**b**) for the tested disc samples at normal load of 20 N.

**Figure 8:**
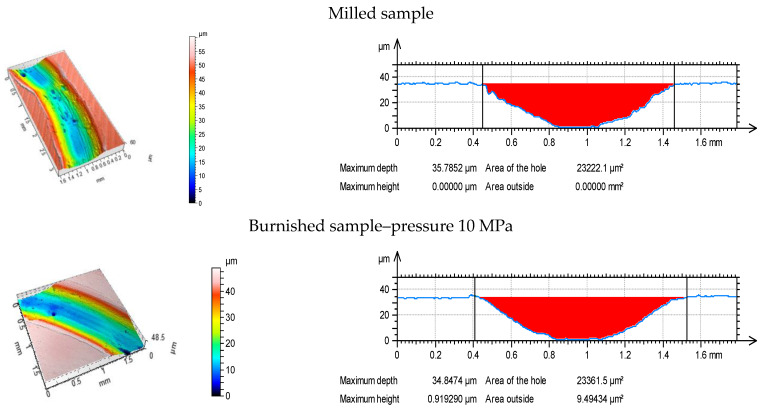

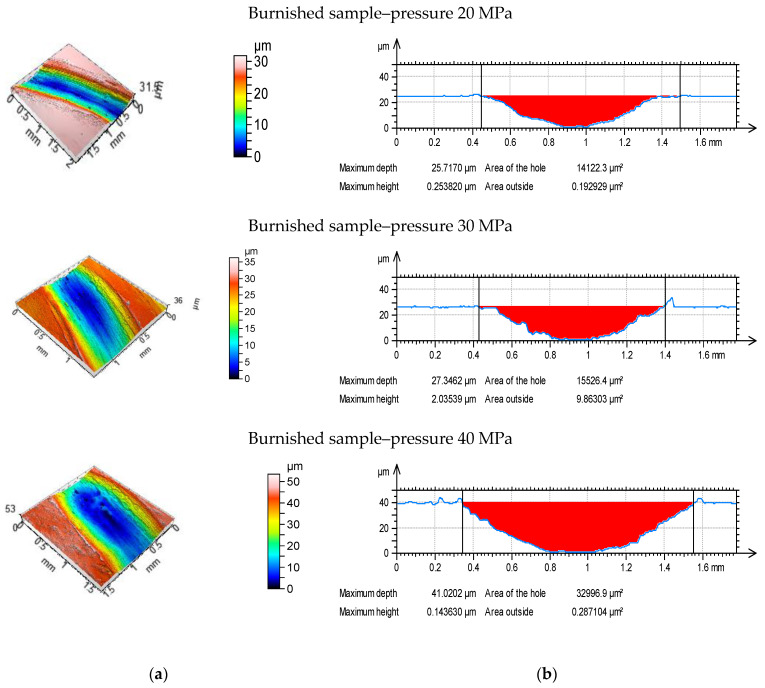
Isometric views (**a**) and profiles perpendicular to wear scars (**b**) for the tested disc samples at normal load of 30 N

Figure 6, Figure 7 and Figure 8 present isometric views and selected profiles orthogonal to wear scars for the tested discs under the various normal loads. Wear had abrasive character. In some cases, the plastic deformations near the edges of wear tracks were observed. 

**Figure 9:**
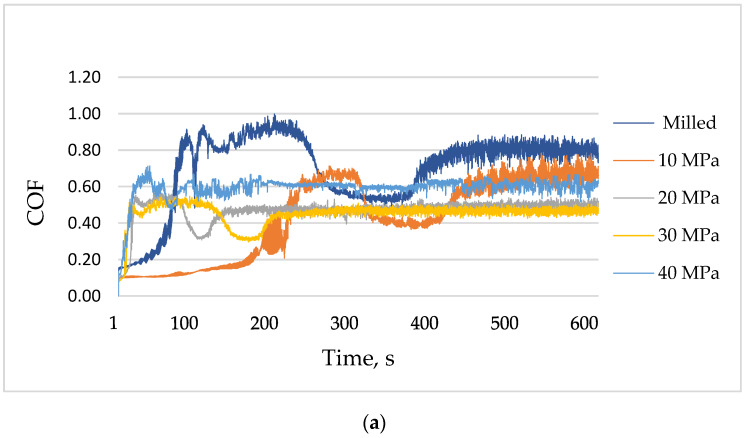

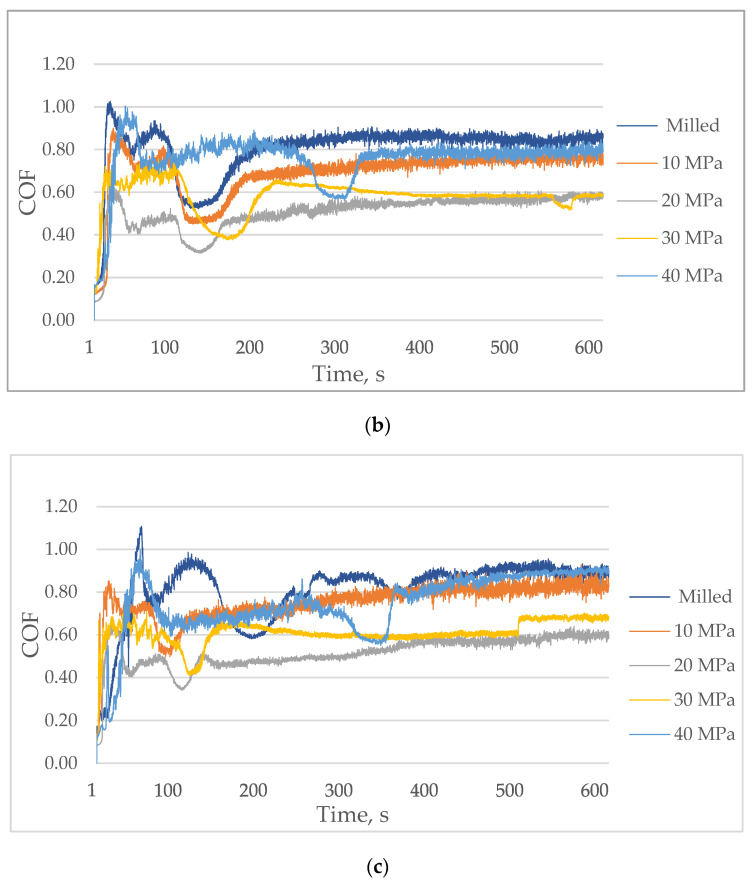
The coefficient of friction versus time for tested assemblies; the normal load was 10 (**a**), 20 (**b**) and 30 N (**c**).

Figure 9 presents runs of the coefficient of friction for various sliding assemblies. 

When the normal load was the lowest (10 N), the volumetric wear increased for small burnishing pressures (10 and 20 MPa) compared to the sliding pair with the milled sample (Figure 5a). Further increases in the burnishing pressure caused decreases in the wear levels. For the smallest load, the highest value and fluctuation of the coefficient of friction occurred for sliding pairs with the milled disc sample. The friction force was stabilized after 450 s and obtained a value near 0.8. Similar behavior was observed for the sliding pair containing the burnished sample with the pressure of 10 MPa; however, in this case, the final value of the coefficient of friction was smaller (near 0.7). When the burnishing pressure was the highest (40 MPa), the final value of the coefficient of friction was also comparatively large (near 0.7), however, the running-in was finished after 100 s, and then, the coefficient of friction obtained the stable value. When the burnishing pressures were 20 and 30 MPa, the friction force was stable after 150 and 200 s, respectively, and the coefficient of friction slowly increased and obtained the final values of about 0.5 (Figure 9a). The best tribological performance (low wear, low and stable friction force) was obtained for the sliding pair containing the sample burnished with the pressure of 30 MPa (Figure 5).

An increase in the normal load to 20 N led to an increase in volumetric wear of milled and burnished samples for the highest pressures (30 and 40 MPa)—Figure 5a. The highest volumetric wear was obtained for the disc burnished with the highest pressure, of 40 MPa. When this pressure was lower, the burnishing process led to a reduction in the frictional resistance, compared to the behavior of the milled disc. The highest reduction was noticed for disc samples burnished with pressures of 20 and 30 MPa. When the normal force was 20 N, the assembly containing milled disc presented initial fluctuations, and after 150 s, the friction force became stable with a slow further increase. The final value of the coefficient of friction was the highest from all analyzed sliding pairs (0.85). However, the final values of the coefficient of friction corresponding to samples burnished with the smallest and highest pressures (10 and 40 MPa, respectively) were a little smaller (about 0.8) compared to that obtained for the sliding pair with the milled disc. The friction force was stable first for smaller burnishing pressures (after 100 s) and later for the highest pressure (320 s). The smallest final resistance to motion was achieved for assemblies with burnished samples with the medium pressures 20 and 30 MPa. In these cases, the final values of the coefficient of friction were near 0.57. However, the friction force runs were different. When the burnishing pressure was 20 MPa, the friction force was stable after 150 s and then slowly increased. When the burnishing pressure was 30 MPa, the friction coefficient obtained the value of 0.65 after 200 s and then slowly decreased (Figure 9b). This behavior was different from those observed for other sliding pairs, for which the coefficient of friction slowly increased with time in the final parts of the tests. An increase in the normal load led to an increase in the coefficient of friction. The best tribological performances were obtained for samples burnished with pressures of 20 and 30 MPa (Figure 5). Wear was proportional to the friction coefficient.

An increase in the normal load from 20 to 30 N caused an increase in volumetric wear of disc samples (Figure 5a). Similar to the medium load, the highest wear was obtained for the disc burnished with the highest pressure (40 MPa). In the other cases, particularly for burnishing pressures of 30 and then 20 MPa, burnishing resulted in a considerable wear reduction. The highest wear decrease, about 2.5 times, was obtained for burnishing pressure of 30 MPa. Similar to operating with medium load, the milled disc and the disc burnished with the highest pressure led to initial fluctuations of the friction force and to the highest final value of the coefficient of friction (about 0.9). When the burnishing pressure was the smallest, the running-in process was finished after 100 s and the friction coefficient slowly increased and obtained the final value about 0.8. For the other sliding pairs, the friction force was stable after 150 s. When the burnishing pressure was 20 MPa, the coefficient of friction slowly increased to 0.6 value. When the burnishing pressure was larger (30 MPa), the coefficient of friction after stabilization decreased, obtained the value of 0.6 and then sharply increased to 0.65 (Figure 9c). However, the friction test runs were similar for the normal loads of 20 and 30 N. An increase in the normal load caused a small increase in the coefficient of friction. When the normal load was 30 N, the best tribological performance was achieved for sliding pairs containing samples burnished with pressures of 30 and then 20 MPa (Figure 5). 

Only for the lowest normal force (10 N) burnishing with the highest pressure (40 MPa) led to a decrease in the coefficient of friction and wear compared to the sliding pair with the milled disc sample (Figure 5). When the normal force was higher (20 and 30 N), ball burnishing with the highest pressure did not cause substantial change of the coefficient of friction and caused an increase in volumetric wear in comparison to the milled disc sample. Ball burnishing yield beneficial tribological results for smaller pressures of 10, 20 and 30 MPa (a reduction in the coefficient of friction and in most cases, a reduction in wear, except for operating at the smallest normal load). The smallest reductions in the coefficient of friction and wear volumes were found for the disc burnished with the smallest pressure of 10 MPa. The best results were achieved for disc samples burnished with the pressures of 20 and 30 MPa. Similar results were obtained when the medium (20 N) and the highest (30 N) normal loads were applied. The biggest fluctuations of the friction coefficient were found when milled and burnished samples with the highest pressure were tested. These fluctuations can be related to the small values of the texture-aspect ratios Str (Table 2). Typically, samples characterized by high wear led also to high coefficients of friction (Figure 5).

A decrease in volumetric wear after ball burnishing can be related to an increase in hardness. The lowest increase in hardness in comparison to the milled specimen was obtained for the disc sample burnished with the smallest pressure (10 MPa)—Figure 4. Probably, this small increase in hardness resulted in comparatively high wear and the coefficient of friction. Burnishing with the higher pressures led to higher and similar hardness. However, the tribological behavior of the sliding pairs having the disc sample burnished with the highest pressure (40 MPa) was the worst. This performance was probably related to the surface topography of this sample (Figure 3e). It is characterized by the smallest Str texture aspect ratio, the smallest peak density and, which is more important, the highest value of the skewness Ssk and the kurtosis Sku (Table 2). The other burnished surfaces were negatively skewed. Probably, the worst tribological performance of the sliding pair containing the disc burnished with the highest pressure resulted from its high positive skewness.

During selection of mating parts, hardness plays the main role. However, surface texture is also substantial. A topography characterized by a high value of the positive skewness should be avoided.

It can be observed from the results of this research that hardness increased as the degree of plastic deformation increased, which confirms the impact of the burnishing pressure in the cold work experienced by surface layers. For the smaller burnishing pressure (10 MPa), a low ratio of plastic deformation occurred, therefore the increase in hardness was small. 

Creation of a surface with the high value of the positive skewness may be caused by too much burnishing pressure (40 MPa) which caused significant plastic deformation, which could contribute to the delamination of thin surface layers. The high value of the burnishing pressure increased the occurrence of metal flakes formed on the surface of the workpiece. 

For the first time, the effect of ball burnishing on the tribological properties of 42CrMo4 chromium–molybdenum alloy steel was studied in dry sliding conditions under pin-on-disc configuration. This arrangement can be used in centrifugal clutch components in claw couplings and quick couplings used in excavators.

The next research will focus on the effect of different burnishing parameters, such as the feed and the speed on the tribological performance of sliding surfaces. Experiments will be carried out in lubricated conditions.

## 4. Conclusions

- Ball burnishing of milled samples led to reductions in amplitude and hybrid parameters and to increases in spatial parameters.
- Due to burnishing, hardness of disc samples increased.
- Ball burnishing led to decreases in the coefficients of friction and in most cases, to reductions in wear volumes of disc samples, compared to the assembly with the milled disc. Similar results were obtained when the medium (20 N) and the highest (30 N) normal loads were applied. In most cases, samples characterized by high wear led also to the high coefficient of friction.
- Due to ball burnishing, volumetric wear was reduced up to 2.2 times, whereas the coefficient of friction to 1.7 times.
- The beneficial tribological results were obtained when the burnishing pressures of 20 and 30 MPa were applied. Burnishing with the highest pressure of 40 MPa yield to the worst results.
- Wear of discs had abrasive character. Due to hardness difference, wear levels of balls were negligible.

## Figures and Tables

**Table 1:** Burnishing parameters.

Burnishing Pressure P MPa	Burnishing Strategy	Burnishing Speed v mm/min	Burnishing Width a mm
10, 20, 30, 40	Spiral [S]	500	0.01

**Table 2:** Selected surface texture parameters of disc samples.

Disc	Milled	Burnished			
Parameters		10 MPa	20 MPa	30 MPa	40 MPa
Sq [µm]	0.710	0.215	0.124	0.317	0.297
Ssk	−0.731	−0.27	−0.154	−0.255	0.910
Sku	2.641	3.593	3.919	3.295	7.309
Sp [µm]	2.067	1.078	0.780	1.680	3.204
Sv [µm]	5.08	1.933	0.816	1.727	3.292
Sz [µm]	7.15	3.012	1.596	3.408	6.582
Sa [µm]	0.597	0.168	0.095	0.250	0.214
Sal [µm]	0.0178	0.121	0.0279	0.0757	0.0282
Str	0.0115	0.406	0.3555	0.4292	0.2201
Sdq	0.104	0.030	0.0279	0.0508	0.0563
Sdr [%]	0.539	0.046	0.0391	0.1289	0.1579
Spd [1/µm^2^]	397.1	485.2	993.7	910.7	354.7
Spc [1/ µm}	88.37	37.77	26.11	47.51	75.93
